# Interdisciplinary Oral Nutrition Support and Supplementation After Hip Fracture Surgery in Older Adult Inpatients: A Global Cross-Sectional Survey (ONS-STUDY) †

**DOI:** 10.3390/nu17020240

**Published:** 2025-01-10

**Authors:** Jack Bell, Ruqayyah Turabi, Sissel Urke Olsen, Katie Jane Sheehan, Ólöf Guðný Geirsdóttir

**Affiliations:** 1 Allied Health Research Collaborative, The Prince Charles Hospital, Chermside, QLD 4032, Australia; 2 Faculty of Food Science and Nutrition, University of Iceland, 102 Reykjavík, Iceland; ogg@hi.is; 3 Department of Population Health Sciences, School of Life Course and Population Sciences, King’s College London, London WC2R 2LS, UK; ruqayyah.turabi@kcl.ac.uk (R.T.); k.sheehan@qmul.ac.uk (K.J.S.); 4 Department of Physical Therapy, College of Nursing and Health Sciences, Jazan University, Jazan 45142, Saudi Arabia; 5 Department of Medical Service, Diakonhjemmet Hospital, 0370 Oslo, Norway; sisselurke.olsen@diakonsyk.no; 6 Bone and Joint Health, Blizard Institute, Queen Mary University of London, London E1 2AB, UK

**Keywords:** hip fractures, hospitals, malnutrition, nutrition risk assessment, nutritional support, oral nutrition supplements

## Abstract

Background: Malnutrition predicts poor outcomes following hip fracture, affecting patient recovery, healthcare performance, and costs. Evidence-based guidelines recommend multicomponent, interdisciplinary nutrition care to improve intake, reduce complications, and enhance outcomes. This study examines global variation in oral nutrition support for older (65+ years) hip fracture inpatients. Methods: A global survey was conducted as part of a broader program to improve interdisciplinary nutrition care. The protocol was based on evidence-based guidelines, reviewed by experts, and piloted for validity. Recruitment used snowball sampling to achieve diversity across income levels, countries, and healthcare roles. Results: The survey (July–September 2023) recruited 308 participants from 46 countries across five global regions. Respondents primarily worked in acute teaching (57.5%) and non-teaching (17.5%) hospitals, representing medical (48.4%), nursing (28.2%), and allied health (17.9%) roles. Findings revealed a global knowledge-to-practice gap in multicomponent nutrition care, across providing high-protein/energy food and fluids (median: “half the time”), post-operative provision of oral nutritional supplements (median: “half the time”) and continuation for one month with assessment (median: “not very often”), and nutritional education (median: “not very often”). Only 17.9% of respondents reported routine provision (“often” and “nearly always or always”) of high-protein/energy food, supplements, and education. Substantial regional variation showed Western Pacific respondents perceiving the lowest provision across multicomponent processes. Interdisciplinary, multicomponent interventions were seen as a potential opportunity requiring further exploration. Conclusions: Major gaps persist in implementing evidence-based, interdisciplinary, multicomponent nutrition care for older adults with hip fractures. A targeted implementation approach is the next step to addressing the knowledge-to-practice gap.

## 1. Introduction

Malnutrition in older people with hip fractures is one of the strongest predictors of morbidity, hospital-acquired complications, length of stay, 12-month mortality, and treatment costs [1,2,3]. It is present in up to half of patients on admission, and without proactive intervention, almost two in three patients are malnourished by (the time of) discharge from the hospital [4]. Consequently, all older adults with a hip fracture should be treated as ‘at risk’ of malnutrition until assessed otherwise, in response to observed high rates of malnutrition, consistently inadequate post-operative intakes, and limited sensitivity and/or specificity of commonly applied screening tools [5,6,7,8].

Systematic reviews, meta-analyses, evidence-based guidelines, and care standards support offering all older hip fracture patients oral nutritional supplements (ONSs) to mitigate nutrition-related complications and improve outcomes [1,5,6,7,8,9,10]. Although definitions vary, ONS can be described as protein, energy-, and nutrient-dense products purposed to increase dietary intake when diet alone is likely to be inadequate to meet nutritional requirements. These may include energy- and protein-enriched drinks (e.g., milk, soy, protein-fortified juice flavors), powders, soups, and/or desserts, such as protein-enriched ice cream or puddings. International guidelines also recommend continuing ONS for malnourished and at-risk patients after discharge [5,6].

However, ONS should not be used in isolation. Practice guidelines [5,11], quality standards [7,8,12], calls for action [13], clinical research, and policy and clinical toolkits [14,15] also consistently advocate for multimodal approaches to nutrition support [16]. These include offering high-quality, enriched, expanded, fortified, or high-protein/energy foods and fluids, snacks, extras, and finger food choices (high-protein/energy choices) [5]; providing nutrition information, education, and/or counseling (nutrition education) for all malnourished patients and those at risk of malnutrition; and coordination of nutrition support processes across healthcare workers and settings [5,6,7,17]. Many of these processes can be delivered by interdisciplinary healthcare workers such as nurses and assistant staff [5,6,7,8]. Multi-, inter-, and transdisciplinary nutrition care approaches can support timely and efficient provision of care and also free up nutrition specialists such as dietitians, nutritionists, and other medical nutrition experts [13,16,18,19].

Barriers to guideline implementation occur at multiple levels, including patients’ health status and motivation, limited nutrition education and awareness among healthcare workers, poorly integrated communication and care processes, and low prioritization of nutrition resources [11,20,21,22]. Although multi-component, interdisciplinary approaches are recommended, evidence suggests that less than half of older inpatients in developed countries routinely receive high-protein/energy diets or oral nutritional supplements (ONSs) post-operatively [23]. Post-discharge ONS use is even less clear, with limited reports indicating that these supplements are rarely prescribed or continued after discharge [23]. Moreover, only about half of malnourished patients appear to receive adequate information regarding their condition and treatment options [23], and the situation in low- and middle-income countries remains largely unknown.

Consequently, significant knowledge gaps persist, including whether older adults with hip fractures are routinely offered high-protein/energy options, ONS during or after hospitalization, or nutrition education to support patient-centered goal setting and shared decision-making. Addressing these gaps is critical for improving adherence and achieving meaningful patient outcomes [24,25,26]. Finally, it remains unclear which healthcare providers are best positioned to support these processes across diverse global settings.

Building the reason for change is the key first step for sustaining and spreading nutrition care improvements in hospitals. Our predefined hypothesis was that the provision of oral nutrition support and supplements to older adults with hip fractures does not align with international recommendations, guidelines, and care standards across global settings. We aimed to explore global variation in oral nutrition support and supplementation processes applied to older (65+) inpatients with hip fractures across global hospital settings. More specifically, the predetermined objectives of this study were to (i) identify whether the provision of oral nutrition support processes is, or is not, aligned to practice guidelines; (ii) capture the variation in oral nutritional support and supplement provision and related processes applied to hospital inpatients with a hip fracture across global settings; and (iii) provide baseline context data to inform and support future initiatives to improve nutrition-related patient outcomes and health system performance after hip fracture.

## 2. Materials and Methods

### 2.1. Design

A global cross-sectional survey, aligned with the STROBE Statement for observational studies, was conducted as a single pragmatic action research cycle within a broader program aimed at improving interdisciplinary nutrition care for older adults with hip fractures worldwide.

### 2.2. Survey Development

The protocol, dataset definitions, and a preliminary survey were developed by the authors, informed by national and international peer-reviewed literature [19], practice guidelines [5,6], and care standards [7]. Face validity was obtained through peer review, piloting, and feedback with purposively sampled interdisciplinary expert clinical academics (by profession and setting) prior to initiating snowball recruitment. A copy of the survey is provided in Supplementary Materials.

### 2.3. Participants and Settings

The survey invited anybody who provided, contributed to, or may have influenced oral nutritional support and supplementation practices for older adults after hip fracture surgery. The survey aimed at capturing maximum diversity and includes medical, nursing, and allied health professionals, policymakers, government and not-for-profit agencies, health administrators; researchers; academics; industry partners; and lower-, middle-, and high-income countries. Participation involved voluntarily completing an anonymous online survey to identify variations in nutrition care practices.

### 2.4. Recruitment

Snowball sampling was employed using invitations disseminated through email lists and social media platforms facilitated by the Fragility Fracture Network (FFN), an organizational gatekeeper with over 9000 members across 100 countries. Participants were encouraged to share study information to expand recruitment beyond FFN membership. Public social media platforms (e.g., X, Facebook, and LinkedIn) and professional networks were encouraged.

### 2.5. Bias

FFN did not enforce regulations, and participation was voluntary and anonymous. The survey landing page provided detailed information emphasizing that the gatekeeper, researchers, and participants sharing the survey could not know who participated. No incentives were offered for survey completion.

### 2.6. Data Sources and Variables

Data were collected via an anonymous, self-administered online survey (Microsoft Forms, Microsoft Corporation, Redmond, WA, USA. Microsoft Office 365. Microsoft, 2024). “Enriched, expanded, fortified, or high-protein/energy menus” were defined in the survey as “These could include additional high-protein or energy fluids or snacks, between-meal/nighttime extras, finger foods, hot meal choices, and the addition of extra oils, fats, or protein (for example, eggs, milk powder, or maltodextrin). For this question, please only consider high-protein or energy foods, fluids, or powders that may be part of an appropriately textured diet/natural foods, not commercially prepared oral nutritional supplements”. ONS were broadly defined as “commercially prepared energy- and nutrient-dense products purposed to increase dietary intake when diet alone is inadequate to meet nutritional requirements. These may include energy- and protein-containing drinks (e.g., milk, soy, protein-fortified juice flavors), powders, soups, and/or desserts”. ONS assessment was defined as “Assessment should consider anthropometric changes (e.g., weight), clinical status, intake adequacy, and other relevant factors to monitor the effects and expected benefits of the intervention and to inform decision-making regarding continuation or cessation of the therapy”. A six-point Likert scale was used (“always or nearly always”; “often”; “about half the time”; “not very often”; “hardly ever or never”; and “I don’t know or don’t want to say”).

An exploratory ‘multicomponent’ care measure was applied to identify the number and percentage of respondents who perceived that high-protein/energy choices, ONS, and nutrition education were offered/provided to older adults with a hip fracture “often” or “always or nearly always”.

Contextual measures collected included the country/nation where the participant primarily worked, world region classification according to the World Health Organization [27], country/nation classification by income [28], participant primary place of work, and healthcare role of participants. An ‘other’ option with free text to enable respondents to provide input where required for demographic contextual measures.

### 2.7. Statistical Analysis

Descriptive statistics were used to summarize all variables. Median and inter-quartile ranges were applied for Likert scale responses. Between-group comparisons were described, and Pearson’s chi-square test was applied for exploratory analysis. No a priori power calculations were performed. Data were analysed using SPSS (IBM SPSS Statistics, Armonk, NY, USA, Version 28.0.1.0).

This study was approved with institutional ethical approval (MRSP-22/23-39291). The survey landing page outlined that participation was voluntary, data collected were anonymous, and withdrawal after submission was not possible due to the anonymized methodology. Participants were required to confirm they had read and understood the information and consented to proceed; otherwise, they could not access the survey.

## 3. Results

### 3.1. Face Validity

Twelve clinical academic experts in fields of nursing (*n* = 2), nutrition/dietetics (*n* = 3), orthogeriatric (*n* = 3), orthopedic surgery (*n* = 3), and physiotherapy (*n* = 1) peer-reviewed and piloted the survey in nine hospitals across six countries. No changes to the survey were required.

### 3.2. Demographics

The survey was piloted (July 2023) and remained open until October 2023. As detailed in Table 1, the survey recruited 308 participants from 46 countries, across 5 of 6 global regions, most of which were high-income countries. Most respondents primarily worked in acute teaching public hospitals, across medical, nursing, and allied health roles.

### 3.3. Alignment of Nutrition Support Processes to Practice Guidelines

**Figure 1:**
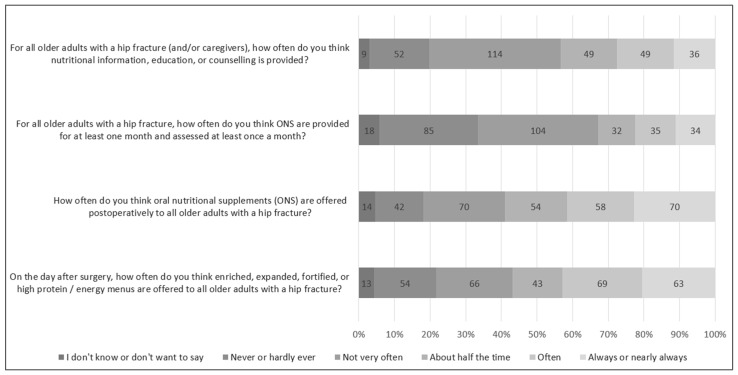
Participant responses to Likert scale questions on nutrition care practices for older adults with hip fractures.

Table 2 and Figure 1 illustrate that most older adults with hip fractures are not perceived to routinely receive care aligned with key evidence-based guidelines and care standards. High-protein foods, fluids, and ONS are reportedly offered to approximately half of older inpatients with hip fractures (Table 2, Figure 1). However, the findings indicate that most patients do not receive ONS for at least one month with at least monthly assessment, nor do they receive nutrition education or counseling. Only 55 respondents (17.9%) perceived that high-protein/energy diets, ONS, and nutrition education were often, nearly always, or always provided to older adults with a hip fracture.

### 3.4. Variation in Oral Nutritional Support Processes Across Settings

Table 3 describes the variation in the routine provision of surveyed care processes across global regions and country income levels. The findings indicate that respondents from the Western Pacific region perceived lower provision of all nutrition support processes compared to other regions. Additionally, the data suggest a positive association between the provision of nutrition education and lower-income countries. Of particular note was the finding that less than one in five respondents perceived that high-protein/energy choices, ONS, and education were provided often or always, regardless of global region or country income.

### 3.5. Providing Baseline Context Data to Inform and Support Future Initiatives

The key objective of this study was to identify which healthcare professionals are perceived as appropriate to offer or provide nutritional support. Descriptive data demonstrate that dietitians/nutritionists and nurses were the individual professional groups most commonly perceived to be appropriate for delivering nutrition support processes (Table 4). No single profession was perceived as capable of routinely providing high-protein/energy choices, ONS, or educational interventions across all settings. However, respondents perceived that, with adequate training, a combination of dietitians, assistants, doctors, nurses, and other allied health professionals could offer these services in virtually all cases.

## 4. Discussion

Our study is the first to highlight a significant and consistent knowledge-to-action gap across 46 countries in 5 of the 6 global regions; oral nutrition support processes appear routinely misaligned to practice guidelines and care standards for older (65+ years) inpatients with hip fracture. We have also uncovered apparent variation in individual nutrition care processes between regions, with respondents from the Western Pacific consistently perceiving the lowest alignment to individual standards of care. It is the first study at scale to identify, from interdisciplinary professionals across these countries and regions, who could offer multicomponent interventions in diverse real-world settings.

### 4.1. Knowledge-to-Practice Gap One—High-Protein/Energy Choices

Our findings demonstrate that high-protein/energy choices are not routinely offered. Clinical trials, evidence-based guidelines, and standards strongly advocate for routine food-first approaches to maintain adequate protein and energy intake, reduce complications, and improve outcomes for older adults with a hip fracture [30,31]. However, clinicians from geographically diverse regions, particularly in the Western Pacific area, perceive that high-protein/energy choices are not routinely offered. Our study is not designed to explore reasons why high-protein/energy foods and fluids were provided only “half the time”, indicating a lack of consistent implementation of these essential nutritional interventions. Further qualitative work is currently under way to elucidate this. In the interim, we have some reflections from clinical experience and other research. For example, it may relate to reliance on families and others to provide food for some countries. Indeed, across many global settings, hospital food services are poorly resourced and not considered a clinical priority. The “opportunity cost” of minor savings in foodservice budgets represents a false economy, as high-quality foodservice systems should be valued as a clinical service [32]. A recent study demonstrated that improved hospital foodservice systems nearly doubled protein and energy intakes, reduced waste, and enhanced patient satisfaction [33]. Another possibility is that interdisciplinary staff are not empowered to prescribe or provide high-protein/energy foods, fluids, and supplements as part of systematized, interdisciplinary improvements from admission; in many settings, access restricted by single professions may delay or prevent access to these [19] Finally, restrictive diets, whether related to social and cultural factors or ‘lifestyle’ diets for weight loss or heart disease, should be deprescribed wherever possible for this patient group [6]. This is particularly important given a recent study showing the increased odds of post-operative delayed mobility, complications, and 12-month mortality associated with a diagnosis of overweight or obese malnutrition in hip fracture [34].

### 4.2. Knowledge-to-Practice Gap Two—ONS

Our studies highlight that oral nutritional supplements are not routinely provided in line with recommendations either post-operatively or for at least one month with regular assessment. Significant regional differences were apparent, with the Western Pacific respondents reporting the lowest perceived rates of ONS provision and assessment. Similarly to the provision of high-protein/energy choices, this also does not appear associated with country economic status. Again, our study is not designed to explore the reasons for this, although findings do suggest it is not simply a result of the cost of supplements. Very low rates of continuation of ONS for one month with assessment beyond the hospital setting may indicate the need for better post-hospital care strategies; however, in many settings, nutrition specialist resources may not be readily available to support this practice [19,35]. Anecdotally, differences in funding models between inpatient and post-discharge settings also influence the ability to prescribe supplements post-discharge. Another potential contributing factor raised by clinicians in practice is that many ONS are milk-based, which may present challenges in regions like the Asia–Pacific where lactose intolerance is more prevalent [36]. However, many of these are low in lactose, and there are many alternatives to the more traditional, milk-based ONS. It is also important to note that our survey included a very broad definition of ONS, and so we surmise that the solution will not be as simple as offering a different drink or other ONS.

Another potential factor contributing to the underuse of both high-protein/energy menus and ONS is the waste generated when these products are not consumed [33,37]. However, it is important to consider that patients are less likely to consume protein- and energy-rich foods, fluids, and supplements if they do not understand their purpose or benefits. For example, other studies suggest that changing flavors and treating ONS as a food rather than a medicine might undermine their utility and instead challenge treating teams to consider whether they are considering malnutrition as a harmful disease with ONS as a medicine to treat it [4]. We strongly recommend that, in addition to simply providing or prescribing supplements, careful attention be given to offering patients clear information on the risks and benefits of high-protein/energy foods, fluids, and oral nutritional supplements (ONSs). This approach is essential to improve adherence and align with informed consent and shared decision-making practices [38].

### 4.3. Knowledge-to-Practice Gap Three—Nutrition Education

Nutritional education was also provided “not very often”, highlighting a gap in patient-centered care and shared decision-making across the global regions. Clear communication and education about the role of malnutrition on recovery and outcomes are essential to ensure adherence and maximize their effectiveness [39]. This raises our most important question: “Why are supplements and high-protein/energy menus perceived to be offered/provided almost twice as much as nutrition education?”. For example, our findings highlight that in Europe, clinicians perceive that high-protein/energy diets and supplements are routinely offered more than half the time. However, nutrition education is perceived to be offered much less often. Consequently, should countries in the region be surprised at reports demonstrating high levels of wastage [37]? This finding is supported by smaller-scale audits in Australia demonstrating that a high proportion of older people with or at risk of malnutrition, or their primary care providers, do not receive diagnostic advice or information [19]. Before changing practice to implement restrictions in offering oral nutrition supplements, perhaps hospitals and treating teams should evaluate whether their patients and staff know why these are being provided or prescribed. Knowledge, perceptions, and social and cultural practices surrounding health and food choices may also come into play, which may detract from the prescription or consumption of ONS. Again, these may be particularly relevant for those with unrecognized overweight or obese malnutrition, type II diabetes, or cardiac disease, or simply an incorrect perception or understanding of what ‘healthy eating’ looks like for older persons living with malnutrition [34]. Rather than simply prescribing or deprescribing menus or ONS, this will require a dedicated and concerted implementation effort to educate patients, healthcare workers, communities, and countries regarding the changes in nutrition requirements over the life span and disease processes [6,16].

Interestingly, our study suggests that patients in lower-income countries are perceived as more likely to receive nutrition information and education than those in high-income countries. Noting the small numbers and rudimentary survey design, the perceived frequency of receiving high-protein/energy menus, ONS, and education trended higher for lower-middle- and upper-middle-income countries. While further research is needed to explore this trend, one possible hypothesis is that scarce resources in these settings are used more thoughtfully, often involving caregivers and family members in the process to ensure that nutrition interventions are both understood and utilized effectively. This is in contrast to higher-income countries, where more readily available foods, fluids, or supplements may be provided without adequate attention to education and support. Further studies are clearly required to explore this. In the interim, we recommend prioritizing patient, caregiver, and healthcare worker engagement and education as a core component of multicomponent, interdisciplinary nutrition care [16].

### 4.4. Opportunity to Improve Outcomes Through Interdisciplinary Care?

All trained dietitians/nutritionists should possess the skills and knowledge to offer or provide fundamental nutritional care processes such as the provision of high-protein/energy foods and fluids, ONS, or education. Our final key finding is that across global settings, dietitians/nutritionists are not always perceived to be available to offer these services. This is not a novel finding, with recent studies demonstrating that there are simply not enough dietitians available to provide timely and appropriate nutrition care to all those at risk [19]. It is highly improbable that across settings, targeted requests to increase the number of dietitians/nutritionists will close this evidence–practice gap [13,40]. As an alternative, interdisciplinary nutrition care has been shown to improve outcomes for older inpatients and is routinely recommended in hip fracture guidelines and care standards as highlighted above [41,42]. Our findings also suggest that enabling interdisciplinary healthcare workers to provide multicomponent care interventions could be an opportunity to improve outcomes. However, translating the perceptions of who ‘could’ offer or provide required practice improvements to actually who ‘does’ these will require a focused and strategic effort within and across individual teams and hospitals, countries, and regions, as well as across clinical, policy, and education domains [4,16]. In many places globally, nutrition care remains either siloed or, at most, multidisciplinary [16]. Rising to this challenge to improve interdisciplinary, multicomponent fundamentals of nutrition care is now a key priority of the Fragility Fracture Network and is embedded in the organization’s strategy, orthogeriatric model, clinical toolkit, and educational program and curricula [43].

### 4.5. Limitations

To capture a snapshot of “real world” care from across global settings, a survey design on participant perceptions was undertaken. This approach is not without limitations. Even with this approach, participation rates for countries, particularly in the Africa, eastern Mediterranean, and Southeast Asia regions, were less than desirable. There were no participants from any low-income countries. Additionally, the open survey design likely attracted respondents with a pre-existing interest in nutrition, which may have led to an overestimation of the extent to which nutritional support is actually provided. While the survey targeted an interdisciplinary group, the low response rate from orthopedic surgeons may reflect underlying engagement challenges that could hinder the implementation of oral nutritional support and ONS. A further limitation is that findings are based on respondents’ perceptions rather than patient-level data, limiting the ability to assess clinical outcomes directly. The rudimentary inferential analyses presented in Table 3 were exploratory in nature, were not adjusted for clinically relevant factors, nor assessed for adequate statistical power. The comparative group for those “often” or “always or nearly always” receiving care included a small number of respondents who “did not know or did not care”. This study does not consider other forms of nutritional supplementation after hip fracture, for example, vitamin D, calcium, or other micronutrients or trace elements [44,45,46]. Finally, the design was focused on those contributing to healthcare; future works are also being undertaken to engage patients and caregivers to explain and explore how they can contribute to co-design efforts to close evidence-to-practice gaps.

Nevertheless, a key strength of the survey was that it clearly articulated, from a diverse population of healthcare workers across 46 countries from 5 global regions, a gap between nutrition recommendations and practice. This provides a strong foundation for a theory-informed nutrition implementation plan across complex healthcare settings that aims to reduce the gap between practice and evidence [47,48,49].

## 5. Conclusions

Malnutrition is a significant predictor of poor outcomes following hip fractures. Despite clear recommendations in practice guidelines and standards, findings suggest fundamental, evidence-based, multicomponent nutrition care for older adults with hip fractures are not routinely provided across global regions. Variations in care provision exist that appear more related to healthcare region rather than economic factors. Clinicians also perceive that in many settings, interdisciplinary healthcare workers could collectively contribute to fundamental nutrition care processes. The next logical step is to undertake a coordinated implementation effort to bridge the evidence-to-practice gap.

## Figures and Tables

**Table 1:** Respondent demographics for 308 participants from across 46 countries.

	*n* (%)
WHO Global Region ^1^	
Africa	0.0 (0.0)
Americas	31 (10.1)
Eastern Mediterranean	2 (0.6)
Europe	131 (42.5)
Southeast Asia	16 (5.2)
Western Pacific	127 (41.2)
Missing	1 (0.3)
Country economic income ^2,3^	
Low-middle and upper-middle income countries	79 (25.6)
Low income	0.0 (0.0)
Low-middle income	33 (10.7)
Upper-middle income	45 (14.9)
High income	228 (74.0)
Missing	1 (0.3)
Primary work setting	
Acute teaching hospital	177 (57.5)
Acute non-teaching hospital	54 (17.5)
Sub-acute or rehabilitation hospital/inpatient center	14 (4.5)
Fragility fracture secondary prevention clinic	11 (3.6)
Primary care setting	8 (2.6)
University/academic center	27 (8.8)
Government agency, policy, or health administration	9 (2.9)
Other	8 (2.6)
Healthcare role	
Medical Doctor ^3^	149 (48.4)
Orthopedic Surgeon	46 (14.9)
[Ortho] Geriatricians	78 (25.3)
Physicians/Rehabilitation Specialist/Internist	15 (4.9)
General Practitioner	5 (1.6)
Other	5 (1.6)
Nursing Professional	87 (28.2)
Allied Health Professional ^3^	55 (17.9)
Dietitians	32 (10.4)
Physical Therapist/Physiotherapist	21 (6.8)
Occupational Therapist	1 (0.3)
Osteopath	1 (0.3)
Research/Academic	16 (5.2)
Government agency, policy, or health administration	1 (0.3)

^1^ WHO (World Health Organization) global regions [27]. ^2^ As defined by the World Bank [28]. ^3^ Sub-categories expressed as percentages of the total.

**Table 2:** Alignment of nutrition care practices with guidelines and standards for older adults with hip fractures.

Survey Questions ^1^	Median (IQR) ^2^
On the day after surgery, how often do you think enriched, expanded, fortified, or high-protein/energy menus are offered to all older adults with a hip fracture?	About half the time (Not Very Often–Often)
How often do you think oral nutritional supplements (ONSs) are offered post-operatively to all older adults with a hip fracture?	About half the time (Not Very Often–Often)
For all older adults with a hip fracture, how often do you think ONS are provided for at least one month and assessed at least once a month?	Not very often (Hardly Ever or Never–About Half the Time)
For all older adults with a hip fracture (and/or caregivers), how often do you think nutritional information, education, or counseling is provided?	Not very often (Not very often–Often)

^1^ Integrating constructs from within key evidence-based guidelines and care standards [5,6,7,29]. ^2^ IQR: interquartile range.

**Table 3:** Variation in perceived frequency of nutritional support processes by global region and country economic income.

	Not Often or Always * *n* (%)	Often or Always *n* (%)	Significance ^α^
**High-protein/energy choices**	175 (57.0)	132 (43.0)	
Americas	17 (54.8)	14 (45.2)	X^2^(3) = 18.010; <0.001
Europe ^1^	60 (45.1)	73 (54.9)	
Southeast Asia	8 (50.0)	8 (50.0)	
Western Pacific	90 (70.9)	37 (29.1)	
Lower-middle and upper-middle income countries	46 (58.2)	33 (41.8)	X^2^(1) = 0.065; 0.799
High-income countries	129 (56.6))	99 (43.4)	
**Oral Nutritional Supplements (ONSs)**	164 (53.4)	143 (46.6)	
Americas	18 (58.1)	13 (41.9)	X^2^(3) = 12.595; 0.006
Europe ^1^	60 (45.1)	73 (54.9)	
Southeast Asia	5 (31.3)	11 (68.8)	
Western Pacific	81 (63.8)	46 (36.2)	
Lower-middle and upper-middle income countries	40 (50.6)	39 (49.4)	X^2^(1) = 0.332; 0.564
High-income countries	124 (54.4)	104 (45.6)	
**Nutrition education**	223 (72.6)	84 (27.4)	
Americas	18(58.1)	13 (41.9)	X^2^(3) = 4.913; 0.178
Europe ^1^	99 (74.4)	34 (25.6)	
Southeast Asia	10 (62.5)	6 (37.5)	
Western Pacific	96 (75.6)	31 (24.4)	
Lower-middle and upper-middle income countries	49 (62.0)	30 (38.0)	X^2^(1) = 6.029; 0.014
High-income countries	174 (76.3)	54 (23.7)	
**High-protein/energy choices, ONS ^2^, and nutrition education**	252 (82.1)	55 (17.9)	
Americas	23 (74.2)	8 (25.8)	X^2^(3) = 5.906; 0.116
Europe ^1^	104 (78.2)	29 (21.8)	
Southeast Asia	13 (81.3)	3 (18.8)	
Western Pacific	112 (88.2)	15 (11.8)	
Lower-middle and upper-middle income countries	61 (77.2)	18 (22.8)	X^2^(1) = 1.715; 0.190
High-income countries	191 (83.8)	37 (16.2)	

For *n* = 307; missing data for *n* = 1. * “Not Often or Always”: Combined categories for “I don’t know or don’t want to say”, “Never or hardly ever”, “Not often”, or “Half the time”. ^α^ Exploratory inferential data; may be subject to Type II error and has not been adjusted for clinically or statistically relevant factors. ^1^ Including one country from the eastern Mediterranean region; ^2^ Either post-operatively or provided for at least one month and assessed at least once a month.

**Table 4:** Number and proportion of interdisciplinary healthcare workers perceived to be appropriate to offer evidence-based nutrition interventions ^1^.

Survey Questions	*n*	%
*With adequate training, who do you think could offer these [enriched, expanded, fortified, or high-protein/energy menus] in most cases? (Tick all that apply)*		
Dietitians/Nutritionists	228	74.0
At least one of the following healthcare workers: ^1,2^	276	89.6
Healthcare Assistants (Medical, Nursing, or Allied Health)	164	53.2
Medical Doctors	163	52.9
Nurses	222	72.1
Other Allied Health Professionals	109	35.4
At least one of the Dietitians/Nutritionists or any other Healthcare Workers	307	99.7
*With adequate training, who do you think could offer these oral nutritional supplements in most cases? (Tick all that apply)*		
Dietitians/Nutritionists	223	72.4
At least one of the following healthcare workers: ^1,2^	282	91.6
Healthcare Assistants (Medical, Nursing, or Allied Health)	150	48.7
Medical Doctors	188	61.0
Nurses	229	74.4
Other Allied Health Professionals	117	38.0
At least one of the Dietitians/Nutritionists or any other Healthcare Workers	306	99.4
*With adequate training, who do you think could assess ONS continuation in most cases? (Tick all that apply)*		
Dietitians/Nutritionists	222	72.1
At least one of the following healthcare workers: ^1,2^	272	88.3
Healthcare Assistants (Medical, Nursing, or Allied Health)	127	41.2
Medical Doctors	173	56.2
Nurses	202	65.6
Other Allied Health Professionals	116	37.7
At least one of the Dietitians/Nutritionists or any other Healthcare Workers	307	99.7
*With adequate training, who do you think could offer nutritional information, education, or counseling in most cases? (Tick all that apply)*		
Dietitians/Nutritionists	243	78.9
At least one of the following healthcare workers: ^1,2^	266	86.4
Healthcare Assistants (Medical, Nursing, or Allied Health)	126	40.9
Medical Doctors	186	60.4
Nurses	215	69.8
Other Allied Health Professionals	122	39.6
At least one of the Dietitians/Nutritionists or any other Healthcare Workers	307	99.7

^1^ Defined as healthcare assistants, medical doctors, nurses, or other allied health professionals. ^2^ Subcategories expressed as percentages of *n* = 308.

## Data Availability

The original contributions presented in this study are included in the article/Supplementary Materials. Further inquiries can be directed to the corresponding author.

## Supplementary Materials

The following supporting information can be downloaded at: https://www.mdpi.com/article/10.3390/nu17020240/s1. PDF version of the Global ONS-Study Survey.
