# Supplementary material for: Interdisciplinary Oral Nutrition Support and Supplementation After Hip Fracture Surgery in Older Adult Inpatients: A Global Cross-Sectional Survey (ONS-STUDY)
Source: Nutrients. 2025 Jan 10;17(2):240. doi: 10.3390/nu17020240 (PMC11767526; doi:10.3390/nu17020240)
Supplement: Supplementary file 1 [file nutrients-17-00240-s001.zip › nutrients-3390505-supplementary.pdf]

Global ONS-STUDY

Oral Nutrition Support and Supplementation afTer hip fracture sUrgery in olDer adults: a global surveY

Ethical Clearance Reference Number: MRSP-22/23-39291

* Required

What is the purpose of the study:
Malnutrition in older people with a hip fracture is a strong predictor of poor outcomes. Oral nutrition support should be offered to all hip fracture patients, not just those who are screened at risk of malnutrition or malnourished, to reduce complications and improve patient and health care outcomes. This survey will help us understand the global variation in recommended oral nutrition support practices for older adults after hip fracture surgery [1-3].

Why have I been invited to take part?
We invite participation from anybody who provides, contributes to, or may influence oral nutritional support and supplementation practices for older adults after hip fracture surgery. This includes medical, nursing and allied health professionals, policymakers, government and not-for profit agencies, health administrators, researchers and academics, industry partners and others.

What will happen if I take part?
If you agree to take part, you will complete a survey anonymously. The survey will ask you questions related to interdisciplinary oral nutrition support and supplementation practices for older adults after hip fracture surgery. The survey will take approximately 5 minutes to complete.

Do I have to take part?
Participation is completely voluntary. You should only take part if you want to and choosing not to take part will not disadvantage you in anyway. If you choose to take part you will be asked to provide your consent. To do this you will be asked to indicate that you have read and understand the information provided and that you consent to your anonymous data being used for the purposes explained. You are free to withdraw at any point during completion of the survey, without having to give a reason. Withdrawing from the study will not affect you in any way. Once you submit the survey, it will no longer be possible to withdraw from the study because the data will be fully anonymous. Please do not include any personal identifiable information in your responses.

Can I forward the survey to others?
This survey applies a snowball sampling approach, initially disseminated by the Fragility Fracture Network (FFN). You may forward the invitation to any of your interdisciplinary colleagues via email or social media. However, please do not forward email contacts to members of the research team or FFN.

What will happen to the results of the study?
Study results are planned for reporting and dissemination, for example via publication in a research higher degree thesis, report, peer reviewed journal and/or presentation at conferences or workshops.

Data handling and confidentiality
This research is anonymous. This means that nobody, including the researchers or those disseminating or forwarding the invitation to participate will be aware of your identity, and that nobody will be able to connect you to the answers you provide, even indirectly. Your answers will nevertheless be treated confidentially and the information you provide will not allow you to be identified in any research outputs/publications. Your anonymous data will be stored on a cloud for 3 years after this project ends.

How is the project being funded?
No funding has been provided for this survey. All works are being provided in kind by members of the research team.

Who should I contact for further information?
For any queries about this study, please contact Jack.Bell@health.qld.gov.au or Ólöf Guðný Geirsdóttir @ ogg@hi.is

What if I have further questions, or if something goes wrong?
If this study has harmed you in any way or if you wish to make a complaint about the conduct of the study you can contact King's College London for further advice and information: Katie Sheehan: katie.sheehan@kcl.ac.uk

Thanks for reading this information sheet and for considering taking part in this research.

1. Volkert, D., et al., ESPEN guideline on clinical nutrition and hydration in geriatrics. Clinical Nutrition, 2019. 38(1): p. 10-47

2. Bell, J.J., et al., Nutritional Care of the Older Patient with Fragility Fracture: Opportunities for Systematised, Interdisciplinary Approaches Across Acute Care, Rehabilitation and Secondary Prevention Settings, in Orthogeriatrics. 2020, Springer, Cham. p. 311-329.

3. Bell, J.J., et al., Systematised, Interdisciplinary Malnutrition Program for impLementation and Evaluation delivers improved hospital nutrition care processes and patient reported experiences – An implementation study. Nutrition & Dietetics, 2021. 78(5): p. 466-475.

1. **By ticking the box below, I confirm that I have read and understood the information provided to me and I agree to take part in this study**. If you do not agree to take part, simply close this window. *

◯ Yes, I consent

2. In which country / nation do you primarily work? *

3. Which of the following best describes where you primarily work? *

- Acute teaching hospital
- Acute non-teaching hospital
- Subacute or rehabilitation hospital / inpatient centre
- Residential care home or hospice
- Subacute or rehabilitation outpatient clinic or centre
- Fragility fracture secondary prevention clinic
- Primary care setting eg. GP, nursing or allied health clinic
- Government agency, policy, or health administration
- University / academic setting
- Healthcare or nutrition-related industry
- Other

4. How is hospital or other inpatient setting predominantly funded? *

- Publically / government
- Privately

5. Which of the following best describes your healthcare role? *

- Allied Health Profession
- Medical doctor
- Nursing profession
- Industry / commercial
- Policy / government / health administration
- Research / academic
- Other

6. What is your allied health profession? *

- ◯ Dietitian or nutritionist
- ◯ Occupational therapist
- ◯ Pharmacist
- ◯ Physical therapist / physiotherapist
- ◯ Psychologist
- ◯ Social worker
- ◯ Speech pathologist
- ◯ Other

7. What is your medical doctor speciality / subspecialty? *

- ◯ General practitioner
- ◯ Geriatrician or orthogeriatrician
- ◯ Othopaedic surgeon
- ◯ Physician
- ◯ Rehabilitation specialist
- ◯ Specialist trainee
- ◯ Other

8. What best describes your nursing profession role/specialty? *

- ◯ Nurse
- ◯ Specialised nurse (eg. consultant, educator, manager, orthopaedic, or practitioner)
- ◯ Other

Please answer the following with regards to **all** older adults who have received hip fracture surgery in your local setting, not just those screened 'at risk' of malnutrition or diagnosed with protein-energy malnutrition.

9. On the day after surgery, how often do you think enriched, expanded, fortified, or high protein / energy menus are offered to **all** older adults with a hip fracture? *

   These could include additional high protein or energy fluids or snacks, between meals/night time extras, finger-foods, hot meal choices, addition of extra oils, fats, protein (for example eggs, milk powder, maltodextrin). For this question, please only consider high protein or energy foods, fluids, or powders that may be part of an appropriately texture diet / natural foods, not commercially prepared oral nutritional supplements.

   - ○ Always or nearly always
   - ○ Often
   - ○ About half the time
   - ○ Not very often
   - ○ Hardly ever or never
   - ○ I don't know or don't want to say

10. With adequate training, who do you think could offer these in most cases? (tick all that apply) *

   - ☐ Dietitians / nutritionists
   - ☐ Medical doctors
   - ☐ Nurses
   - ☐ Other allied health professionals
   - ☐ Health care assistants (medical, nursing, or allied health)
   - ☐ Other

11. How often do you think oral nutritional supplements (ONS) are offered postoperatively to **all** older adults with a hip fracture? *

For this survey, ONS are defined as commercially prepared energy and nutrient-dense products purposed to increase dietary intake when diet alone is inadequate to meet nutritional requirements. These may include energy and protein containing drinks (eg. milk, soy, protein-fortified juice flavours), powders, soups, and/or desserts.

- ◯ Always or nearly always
- ◯ Often
- ◯ About half the time
- ◯ Not very often
- ◯ Hardly ever or never
- ◯ I don't know or don't want to say

12. With adequate training, who do you think could offer these in most cases? (tick all that apply) *

- ☐ Dietitians / nutritionists
- ☐ Medical doctors
- ☐ Nurses
- ☐ Other allied health professionals
- ☐ Health care assistants (medical, nursing, or allied health)
- ☐ Other

13. For **all** older adults with a hip fracture, how often do you think ONS are provided for at least one month **and** assessed at least once a month? *

Assessment should consider anthropometric changes (eg. weight), clinical status, intake adequacy and other relevant factors to monitor the effects and expected benefits of the intervention and to inform decision making regarding continuation or cessation of the therapy.

- ◯ Always or nearly always
- ◯ Often
- ◯ About half the time
- ◯ Not very often
- ◯ Hardly ever or never
- ◯ I don't know or don't want to say

14. With adequate training, who do you think could assess ONS continuation in most cases? (tick all that apply) *

- [ ] Dietitians / nutritionists
- [ ] Medical doctors
- [ ] Nurses
- [ ] Other allied health professionals
- [ ] Health care assistants (medical, nursing, or allied health)
- [ ] Other

15. For **all** older adults with a hip fracture (and/or caregivers), how often do you think nutritional information, education, or counselling is provided? *

Nutritional information and education should include individualised provision of information regarding current and predicted nutritional status, principles of shared decision making and informed consent, and individual counselling including the potential benefits and risks of treatment versus no treatment options, for example whether to accept oral nutritional supplements offered.

- ○ Always or nearly always
- ○ Often
- ○ About half the time
- ○ Not very often
- ○ Hardly ever or never
- ○ I don't know or don't want to say

16. With adequate training, who do you think could provide this information in most cases? (tick all that apply) *

- [ ] Dietitians / nutritionists
- [ ] Medical doctors
- [ ] Nurses
- [ ] Other allied health professionals
- [ ] Health care assistants (medical, nursing, or allied health)
- [ ] Other

This content is neither created nor endorsed by Microsoft. The data you submit will be sent to the form owner.

Microsoft Forms
